# Local Order and Dynamics of Nanoconstrained Ethylene-Butylene Chain Segments in SEBS

**DOI:** 10.3390/polym10060655

**Published:** 2018-06-11

**Authors:** Michele Mauri, George Floudas, Roberto Simonutti

**Affiliations:** 1 Department of Materials Science, University of Milano Bicocca, via R. Cozzi 55, 20125 Milan, Italy; michele.mauri@unimib.it; 2 Department of Physics, University of Ioannina, 45110 Ioannina, Greece and Max Planck Institute for Polymer Research, Ackermannweg 10, D-55128 Mainz, Germany; gfloudas@uoi.gr

**Keywords:** block copolymers, polystyrene-*b*-poly (ethylene-*co*-butylene)-*b*-polystyrene, X-ray diffraction, ^13^C Magic Angle Spinning NMR, ^1^H Time Domain NMR, dielectric spectroscopy, rotator phase, mesophase

## Abstract

Subtle alterations in the mid-block of polystyrene-*b*-poly (ethylene-co-butylene)-*b*-polystyrene (SEBS) have a significant impact on the mechanical properties of the resulting microphase separated materials. In samples with high butylene content, the ethylene-*co*-butylene (EB) phase behaves as a rubber, as seen by differential scanning calorimetry (DSC), time domain (TD) and Magic Angle Spinning (MAS) NMR, X-ray scattering at small (SAXS), and wide (WAXS) angles. In samples where the butylene content is lower—but still sufficient to prevent crystallization in bulk EB—the DSC thermogram presents a broad endothermic transition upon heating from 221 to 300 K. TD NMR, supported by WAXS and dielectric spectroscopy measurements, probed the dynamic phenomena of EB during this transition. The results suggest the existence of a rotator phase for the EB block below room temperature, as a result of nanoconfinement.

## 1. Introduction

Block copolymers, constituted by rigid and soft and chemically unlike blocks, have important applications as “thermoplastic elastomers” (TPEs) [1]. An important class of TPEs has the rigid part constituted by a styrenic block, while the mobile part is constituted by aliphatic chains. A common synthetic route is by anionic block polymerization of styrene with isoprene or butadiene, producing styrene-isoprene block copolymers (SIS) and poly(styrene-butadiene-styrene) (SBS), respectively, followed by a catalytic hydrogenation that is selective for the unsaturated aliphatic part. The resulting polystyrene-*block*-poly(ethylene-*co*-but-1-ene)-*block*-polystyrene triblock copolymers (SEBS) have enhanced mechanical properties and increased resistance to UV exposure. SEBS displays a morphological behavior typical of block copolymers [2,3,4] depending upon several variables, such as the length ratio between the immiscible blocks, and the amount and distribution of butyl repeat units (i.e., ethyl side chains) in the mid-block. Due to their relevance for industrial applications, various aspects of SEBS materials were studied, including the morphology [5], the diffusion of small molecules in the EB phase [6], and the reorganization of the phase structure following mechanical stress [7] or nanometric confinement [8]. Most studies concerned the order to order transitions at high (>430 K) temperature that are now well understood. The mobile (elastomeric) phase was also investigated by several papers [9,10,11,12] without reaching such conclusive results. Several authors highlighted the presence of a broad endothermic transition in the calorimetric curves of SEBS with low butyl content. They attributed this to crystallization of the ethylene-rich regions of the EB blocks with few direct proofs [13]. A peculiarity of polymers is that several modes of phase organization exist between melt and crystal, including mesophases, interfaces and interphases. Mesophases, stable only in small ranges in the case of bulk polymers, can be present in a more extended range in confined systems [14] or in the presence of side chains [15]. Nuclear magnetic resonance (NMR) is a particularly appropriate technique for the investigation of these phases [16,17,18], since it directly probes motional mechanisms and characterizes disordered states that are difficult to recognize unambiguously by X-ray diffraction (XRD) [19]. Recent developments [20,21,22] specifically suggest using a ^1^H time domain NMR (TD-NMR) for characterizing polymer dynamics along a wide range of temperature. In particular, measurement of the transverse relaxation time ^1^H *T*_2_ by Hahn echo [23] has become a mainstay in the study of chain dynamics in rubbers and other polymers [20]. The limitations of this approach were highlighted and new alternatives were explored [24], including the Baum Pines sequence that stimulates and measures multiple quantum coherences (MQ), which is used for the study of polymers [25], rubbers [26,27], and gels [28] as well as polymer melts, as exemplified by Spiess and co-workers [29].

Herein, we characterize the dynamics of the ethylene-*co*-butylene mid block chains using advanced TD-NMR, in addition to more established techniques (Dielectric Spectroscopy, DSC), to describe local aggregations and constricted mobility as defined by the nanostructured morphology. Our results demonstrate that the dynamic features are coupled with the nanodomain morphology and local ordering obtained, respectively by small-angle and wide-angle X-ray scattering. The EB phase in SEBS is influenced by two contrasting factors: the presence of a varying amount of ethyl side chains incompatible with PE crystallization, and the stabilizing effect of tethering on both sides. A multitechnique approach allowed determining the nature of the peculiar phases obtained in the presence of a limited amount of side chains, and the return to a rubbery behavior when the ethyl side chains become exceedingly abundant

## 2. Materials and Methods

### 2.1. Materials

**Scheme 1:**
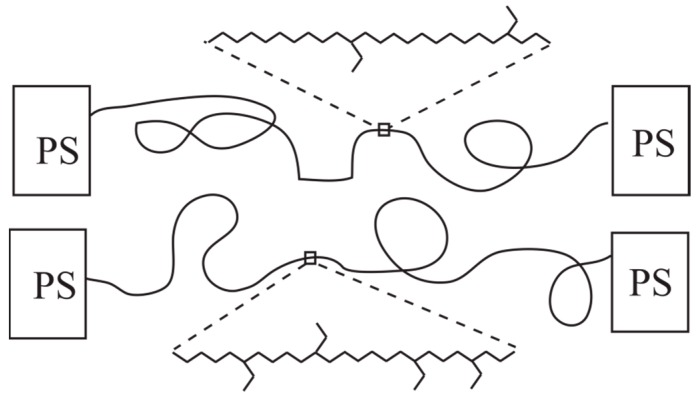
conceptual description of the polystyrene-*block*-poly(ethylene-*co*-but-1-ene)-*block*-polystyrene triblock (SEBS) copolymers, with the LB on top and the HB on the bottom. The different amount of side chains in the mid block is highlighted.

Six different qualities of polystyrene-*block*-poly(ethylene-*co*-but-1-ene)-*block*-polystyrene (SEBS) were kindly provided by Kraton Performance Polymers (Houston, TX, USA) and used without further purification. All are triblock copolymers with polystyrene blocks surrounding a central block of poly(ethylene-*co*-butylene). The butyl comonomers provide the central block of ethyl side chains, samples with lower side chain content are labeled LB1 and LB2 while those with high side chain content are named HB1, HB2 and HB3, Scheme 1. Additionally, we had a single sample of polystyrene-*block*-poly(ethylene-*co*-butylene-*co*-styrene)-*block*-polystyrene, with styrene monomers also included into the central block. This sample is labeled LBS.

To provide uniform specimens for the NMR, X-ray diffraction, dielectric spectroscopy, and calorimetric studies, all the polymers were cast from toluene, a selective solvent for the styrene block [5]. Solution cast films (25–100 μm) were prepared by very slow evaporation (several days) of a 2% weight solution.

### 2.2. X-ray Diffraction

Both wide-angle and small-angle X-ray scattering (WAXS/SAXS) measurements have been performed from cast films of the copolymers using a pinhole collimator. In WAXS, Osmic confocal optics were used for monochromatic X-ray beam (*λ* = 0.15418 nm). The detection system was a MAR345 Image Plate Area Detector (Marresearch GmbH, Norderstedt, Germany) and the sample-to-detector distance was 22.23 cm. Variable temperature measurements were made from 223 to 353 K on heating and on subsequent cooling in steps of 10 K.

SAXS measurements were made with a Rigaku RA-Micro 7 Desktop Rotating Anode X-Ray Generator (Rigaku, The Woodlands, TX, USA) with a maximum power of 800 W and brightness of 18 kW/mm^2^ (operated at a tube voltage of 40 kV and tube current of *I* = 10 mA (400 W)) utilizing a Cu target. The sample-to-detector distance was set at 1.58 m and the same films were employed as in WAXS. 2-D images were obtained with a two-dimensional detector (Bruker, Billerica, MA, USA) with 1024 × 1024 pixels. Measurements of 1800 s long (following another 1800 s of annealing) were made for different temperatures in the range 225 < *T* < 473 K in 10 K intervals on heating and subsequent cooling. In both SAXS/WAXS the recorded 2-D scattered intensities were investigated over the azimuthal angle and are presented as a function of the modulus of the scattering vector *q* (*q* = (4π/*λ*)sin(2θ/2), where 2θ is the scattering angle).

### 2.3. Differential Scanning Calorimetry

Differential Scanning Calorimetry (DSC) analyses were performed by using a Mettler-Toledo DSC 821e equipped with STAR^e^ software version 11.0 (Greifensee, Switzerland) and liquid N_2_ temperature regulation, using standard 40 μL crucibles.

### 2.4. Size Exclusion Chromatography

Size Exclusion Chromatography was performed with a Waters 1515 Isocratic HPLC pump using solutions of polymer in THF with a concentration of 2 mg/mL (0.2 wt %). Solubility at this concentration was very good. THF flow was 1 mL/min and temperature was maintained at 298 K. Separation was provided by four Styragel columns (HR2, HR3, HR4, HR5) and detection was provided by a Waters 2414 refractive index detector. A calibration with polystyrene standards (Sigma-Aldrich, Saint Louis, MO, USA) was used.

### 2.5. High Resolution Liquid State NMR

Characterization of the polymers was performed also in a liquid state using a Bruker Avance 500 system (Karlsruhe, Germany) with 500.13 MHz proton resonance and 125.70 MHz ^13^C resonance. All samples are soluble in CDCl_3_.

### 2.6. Magic Angle Spinning NMR

Solid-state ^13^C NMR spectra were run at 75.5 MHz on a Bruker Avance 300 instrument. The samples were spun at 15 kHz, and ramped-amplitude cross-polarization (RAMP-CP) [30] transfer was applied.

### 2.7. Dielectric Spectroscopy

Dielectric measurements were made with a Novocontrol Alpha frequency analyzer under “isochronal” conditions (at a frequency *f* = 1.33 × 10^5^ Hz in the caption of Figure 7) with a rate of 2 K/min. During these runs the complex dielectric permittivity ε* = ε′ − iε″, where *ε*′ is the real and ε″ is the imaginary part, was recorded as a function of temperature *T*.

### 2.8. Time Domain NMR

Experiments were carried out on a Bruker minispec mq20 spectrometer operating at around 0.5 T static magnetic field, with proton resonance of 19.65 MHz, pulse length for the 90° between 2.75 and 3 μs, phase switching time around 2.1 μs and receiver dead time τ set at 14 μs. Most experiments were well accumulated with less than 128 scans. Recycle delays of 1–2 s were sufficient. Rigid fraction *f_r_* was studied by implementing the Magic Sandwich Echo (MSE) refocusing block [31] optimized for quantitative detection [32]. Direct decomposition of the FID provides *f_r_* through the following equation:

(1) $$\frac{FID\left(t\right)}{FID\left(0\right)}=f_{r}exp{\left(-\left(\frac{t}{T_{2r}^{*}}\right)\right)}^{2}+\left(1-f_{r}\right)exp{\left(-\left(\frac{t}{T_{2m}^{*}}\right)\right)}^{v}$$

where $T_{2x}^{*}$ are apparent longitudinal relaxation times for the rigid (*r*) and mobile (*m*) phases, respectively, and *ν* is a coefficient that parameterizes a deviation of the more slowly relaxing signals from purely exponential shape.

We then performed the study of chain dynamics by Hahn Echo [33], but also with the Baum Pines sequence, as implemented by Saalwächter [21] and later refined with the use of Tichonov regularization [34]. The experiments consist in the detection of multiple quantum (MQ) coherence buildup as a function of the duration of the excitation block of the sequence. This buildup is then associated to a distribution of residual dipolar couplings (*D*_res_) that describe the local interactions within the material: regions where the polymer motion is more constrained present higher values of this observation. Both chemical and physical crosslinks influence the *D*_res_, and while most current applications focus on assessing chemical crosslinking of vulcanized rubber [27], the effect of physical confinement on polymer dynamics can also be measured.

## 3. Results

### 3.1. Polymer Characterization

Molar weight and polymer microstructure were assessed in our lab by SEC and high resolution liquid state ^1^H and ^13^C NMR respectively as shown in Table 1.

Chromatographic data indicate a sharp and monomodal *M*_n_ distribution, with PDI values close to unity, as shown in Table 1. The composition on styrene is easily calculated by quantitative ^1^H NMR, since the peaks associated to aromatic and aliphatic regions do not overlap. ^13^C High Resolution NMR spectra (exemplified in supporting information) were assigned following the work of Quirk et al. [35], distinguishing the side chain carbons associated to the butylene fraction. By subtracting the backbone carbons associated with the known amount of styrene, we calculated the butylene content relative to the EB block alone.

The HB samples display a lower relative amount of PS but larger *M*_n_. As a result, most samples have a very similar PS block size, with a mass of about 10 kDa corresponding to around 100 repeating units each. The LBS sample has a significant amount of styrene units in the mid block; the total molar fraction of styrene is almost 28%, more than one third of which is included in the mid block. LB1 and LB2 have identical composition and are differentiated only by the average chain length. The same holds for HB1 and HB2. HB1 and HB3 instead share the same molecular weight and comonomer composition, but have a different quantity of ethylene in the EB phase.

### 3.2. Film Characterization

**Figure 1:**
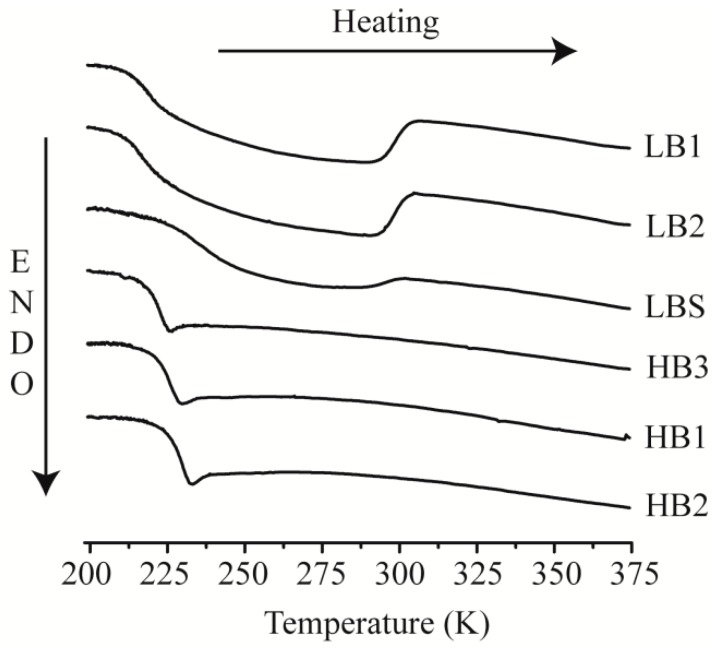
Overview of the Differential Scanning Calorimetry (DSC) traces of all samples during heating from 200 to 375 K at 10 K/min.

Calorimetric measurements on SEBS films are shown in Figure 1 and then summarized in Table 2. The samples with high butylene content display a typical glass temperature around 220 K. Low butylene content SEBS display an extremely broad endothermic transition with onset at around 215 K, extending to 300 K.

This feature was reported for the first time by Sierra et al. [9] while studying SEBS with a broad variation of butyl content synthesized in their laboratory. In LB1 and LB2 this feature is more pronounced than in sample LBS where the onset is shifted to higher temperatures and the global enthalpy is reduced. The enthalpy associated to the transition in the LB samples is in the range of 30–50 kJ/g. The only component effectively capable of crystallization consists of the strands of ethyl units contained in the EB phase. By considering that PS is around one third of the total mass, and that the melting enthalpy of idealized PE crystal phase is 293 kJ/g [36], we estimate the EB part contains 20 ± 5% crystalline PE.

**Figure 2:**
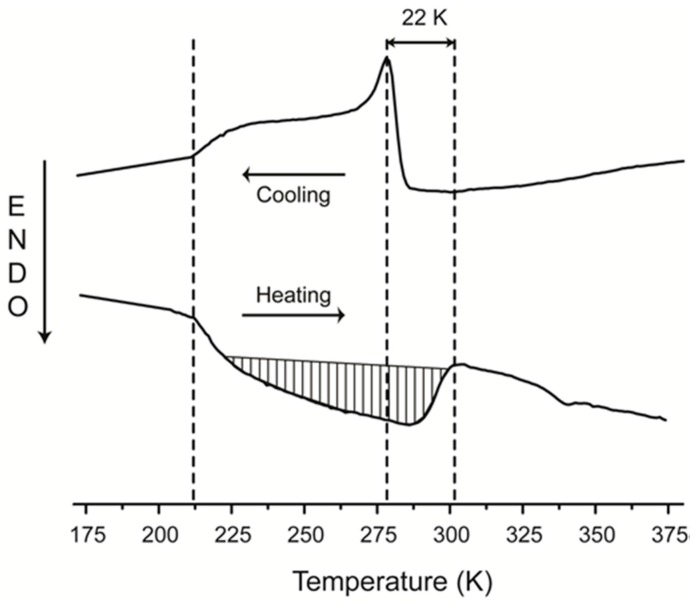
Heating and Cooling DSC traces of LB1, a sample with low butyl content, at 10 K/min.

The DSC curves acquired during cooling of HB samples also display a glass temperature (supporting information). LB samples have a different behavior, as seen in Figure 2: the thermogram acquired during cooling displays a peak shifted by around 20 K relative to the end of transition during heating, being reminiscent of the crystallization peak in crystallizable polymers.

**Figure 3:**
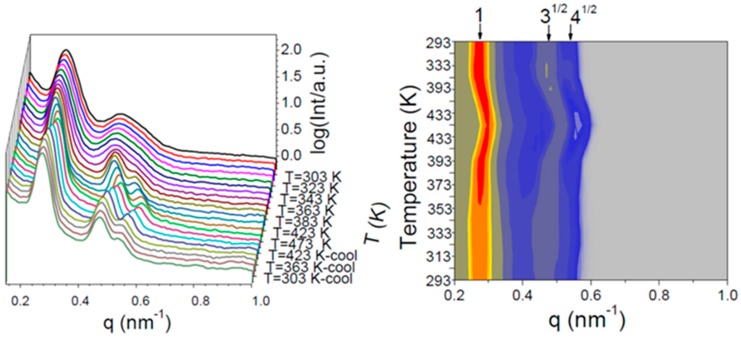
(**Left**) X-ray scattering at small (SAXS) intensity profiles for HB1 shown for different temperatures on heating and subsequent cooling. Annealing causes reversible and irreversible changes on the nano-domain morphology. (**Right**) The same data are now shown as intensity contour plots. The dependence of the first and higher order reflections following annealing at 473 K indicates shrinkage upon heating. Arrows indicate peaks with ratio 1:3^1/2^:4^1/2^ relative to the first peak corresponding to 293 K.

**Figure 4:**
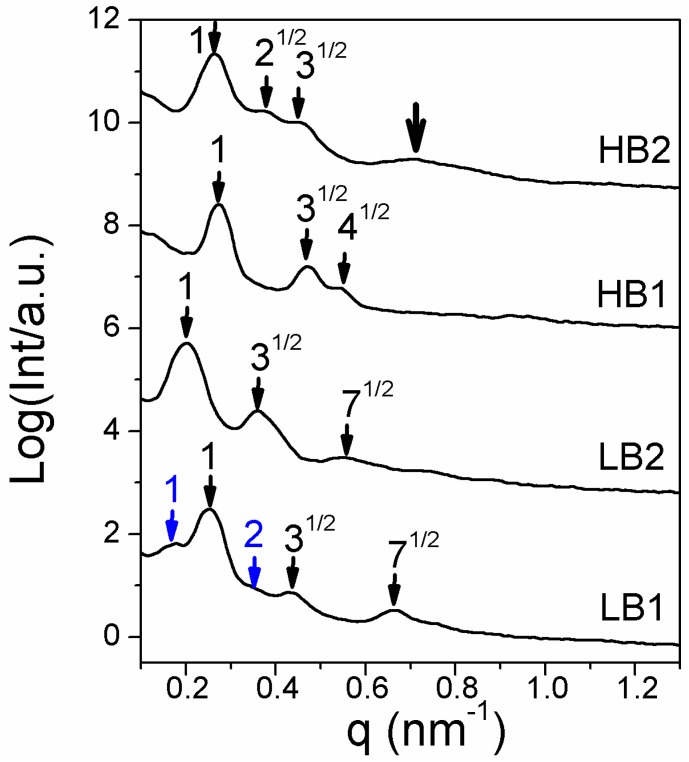
SAXS intensity profiles measured at 303 K following annealing at 473 K. The thin arrows represent the first- and higher order reflections from the nanodomain morphology. The thick arrow in HB2 gives the intra-particle scattering peak.

The results from the SAXS measurements are compared in Figure 3 and Figure 4. In all cases the as received block copolymer films prepared by casting from toluene solutions display features that are characteristic of systems that are away from equilibrium, which broad reflections and continuously evolving reflections with temperature/annealing clearly demonstrate. Figure 3, gives an example of the annealing effects on HB1. The very broad higher order reflections break in well resolved peaks by annealing at 473 K. On subsequent cooling the nanodomain morphology did not change, suggesting that the structure obtained by slow cooling is at equilibrium. Another interesting feature is the temperature dependence of the first (and higher) order reflections. On first heating the peak moves to higher wavevectors (shorter distances) with an onset at the PS glass temperature up to 473 K; on subsequent cooling it shows the opposite temperature dependence. This feature of negative thermal expansion is the characteristic of the rubbery state of the material.

Returning to the nanodomain structures (Figure 4), we find that an initially lamellar nanodomain morphology (with peak positions relative to the primary peak with ratios 1:2:3) in the as cast films transforms to hexagonally close packed cylinders (hcpc) in LB2 and HB1 with relative peak positions at 1:3^1/2^:7^1/2^ (LB2) or at 1:3^1/2^:4^1/2^ (HB1) following slow heating and annealing at 473 K. Similar measurements were performed for all samples, and the resulting nanodomain spacing and structures are presented in Table 2.

For the LB1, however, we found that the nanodomain morphology in addition to a majority of hcpc contains also a mixture of a lamellar phase. This finding is in agreement with an earlier observation in the same copolymer showing a transformation from lamellar to hcpc morphology on annealing at 441 K for 120 min. However, in the present case, heating to 473 K for 30 min and subsequent cooling did not completely eliminate the initial lamellar structure. This is suggestive of a complex time-temperature relation for the equilibration of the nanodomain morphology.

The nanodomain morphology of the sample with the lowest PS content (HB2) instead presents reflections at relative positions 1:2^1/2^:3^1/2^ compatible with a cubic structure (a bcc morphology has peaks at 1:2^1/2^:3^1/2^:2:5^1/2^:6^1/2^:7^1/2^). Even if not all reflections are visible, the assignment of a bcc structure—among the different cubic structures—can be made based on the known PS volume fraction, *f*_PS_ and the peak due to intra-particle interference (thick arrow in Figure 4). For a bcc lattice, the radius of PS spheres can be obtained by *R*_PS_ = (*f*_PS_*d*_0_^3^/π3^1/2^)1/3 where *f*_PS_ = 0.1, *d*_0_ = *d*(3/2)^1/2^ is the nearest neighbor distance between spheres and *d* = 2π/q* giving *R*_PS_ = 7.7 nm. On the other hand, the estimation of the radius of PS spheres can be made as *R*_PS_ = 5.764/*q*_max_, where *q*_max_, is the broad peak due to intra-particle interference. Using *q*_max_~0.71 nm^−1^ we obtain *R*_PS_ = 8 nm, in good agreement with our earlier estimate.

### 3.3. Magic Angle Spinning NMR

The results presented above indicate the presence of different domains but do not indicate the compositional purity of each. To verify that the detected periodicity corresponds to the chemical separation of the blocks, samples were also characterized using CP-MAS and high power decoupling (HPDEC) ^13^C NMR. The *T*_1_ relaxation time of carbon nuclei is in the range of tens of seconds for nuclei associated to rigid phases. By using a short recycle delay in HPDEC conditions, the recovery of magnetization associated to the rigid phase is negligible and the technique is selective to the mobile phases. Signal generation in CP-MAS experiments is instead based on polarization transfer from proton nuclei, a process that is more efficient in the presence of strong dipolar couplings typical of rigid phases.

**Figure 5:**
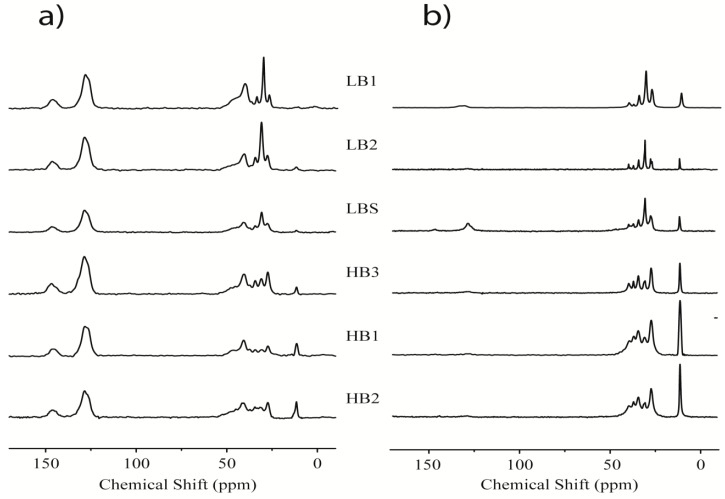
(**a**) CP-MAS and (**b**) HPDEC ^13^C NMR spectra of all SEBS samples at 298 K.

Experiments were performed at 298 K, at the upper bound of the DSC transition and are presented in Figure 5. Carbon NMR measurements easily distinguish the signals associated to the styrene fraction (128 and 145 ppm) [37] from those of the aliphatic fraction around 30 ppm.

The most striking feature of the presented spectra is that all CP-MAS spectra; (a) evidently present aromatic functional groups, but when the mobile part is selected by HPDEC (b) those signals associated to PS blocks are practically absent. The exception is LBS, where the styrene units are copolymerized in the mid block and share the higher mobility of the EB. The carbon NMR measurements confirm there is no mixing between the PS and EB blocks, unless in the sample where styrene is purposefully copolymerized in the mid block, and show very little evidence of an extended interphase. Thus, in further discussions we can define a mobile phase entirely composed of EB, and reason in terms of EB dynamics.

Analysis of the aliphatic range in (a) indicates the backbone methylene carbons (30 ppm) are the most intense signals in LB1 and LB2, while in HB samples the signals around 26.7 and 11.1 ppm, representing the two side chain carbons, are prevalent. These results are in agreement with the relative ethylene and butylene content. In the HPDEC spectra, the highly mobile methyl groups at 11.1 ppm are further enhanced, and confirm the high amount of butyl units in HB samples.

### 3.4. Wide-Angle X-ray Diffraction (WAXD)

**Figure 6:**
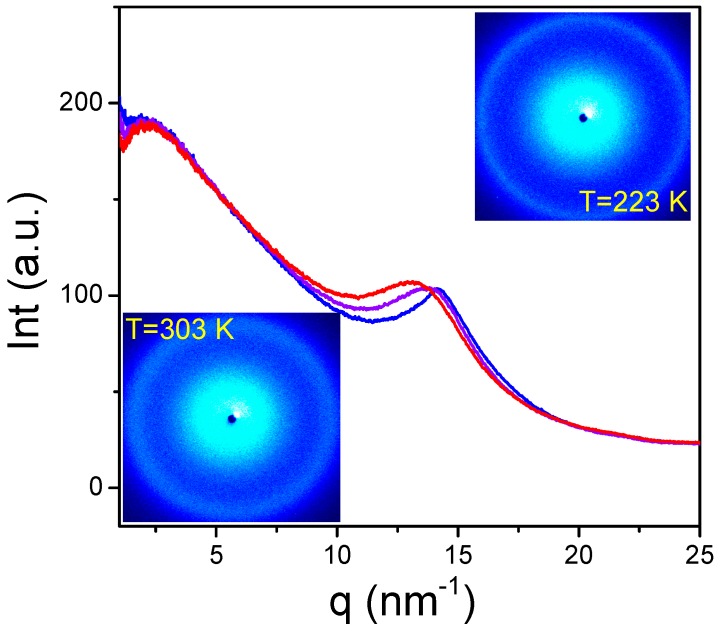
Wide-Angle X-ray Diffraction (WAXD) intensity profiles for LB2 shown for selected temperatures: 223 K (blue), 283 K (magenta), and 353 K (red). 2D images are also shown at two temperatures (lower): 303 K and (upper): 223 K. In both representations, there is a noticeable narrowing of the reflection at ~14.3 nm^−1^.

The WAXD measurements on the LB1 and LB2 copolymers performed at low temperatures provide additional information on the local structure within the nano-domains. The WAXD intensity profiles for LB2, shown in Figure 6, display broad peaks centered at about 2 and 14.3 nm^−1^ corresponding to distances of 3.1 and 0.44 nm, respectively.

These are characteristic of amorphous systems and are known as van der Waals peaks [38,39]. However, when cooled to the temperatures corresponding to the lower bound of the broad thermal transition, respectively 223 and 243 K, the peak at 14.3 nm^−1^ sharpens significantly, suggesting the ordering/weak-crystallization of the EB part. At the same time there is increased intensity at lower *q* values. We note here that in PE the 110 reflection is at 15.4 nm^−1^ for the orthorhombic unit cell [40]. Thus, we can define weak crystallization as the formation of highly distorted crystals that produce WAXD intensity measurably different from the pure amorphous system but still very far from the well formed PE crystals described in literature.

In summary, the X-ray analyses have shown non-equilibrium structures in the as-cast films that reach the equilibrium nano-domain morphologies only after heating to 473 K and annealing for long times. WAXD revealed that by cooling the copolymers LB1 and LB2 to low temperatures corresponding to the beginning of the broad thermal transition, the EB part starts to order and exhibits weak crystallization.

### 3.5. Dielectric Spectroscopy

**Figure 7:**
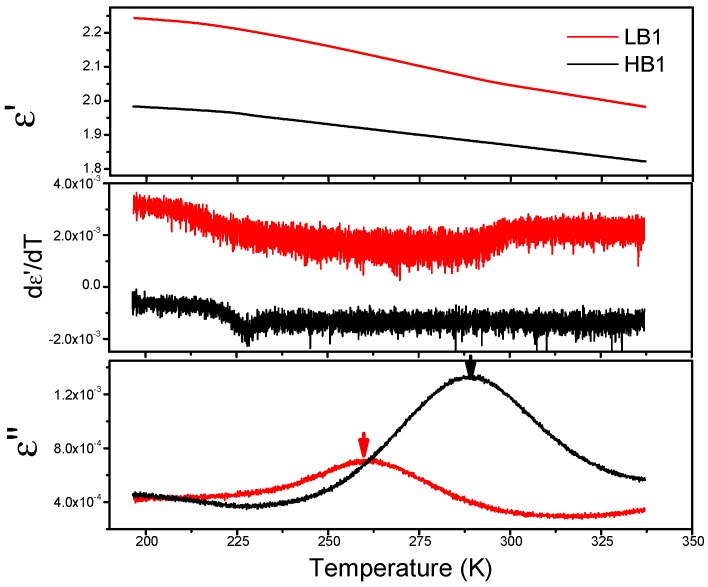
Dielectric permittivity (top), derivative of the dielectric permittivity with temperature (middle) and dielectric loss (bottom) of LB1 (red line) and HB1 (block line) obtained during the second heating run with a rate of 2 K/min at a frequency of 1.33 × 10^5^ Hz. The derivative of LB1 has been shifted vertically for clarity. The arrows in the derivative representation provide the temperature of the segmental relaxations at the chosen frequency.

Dielectric measurements under isochronal conditions were performed on two copolymers. Results from the second heating run are shown in Figure 7. During these runs the complex dielectric permittivity ε* = *ε*′ − i*ε*″, was recorded as a function of temperature *T*. The derivative of dielectric permittivity with temperature is especially informative and has been used in the past to identify phase transitions in several materials [41,42]. For HB1 it shows a step at about 230 K, at the same temperature as the DSC glass temperature. For LB1, the trace is also reminiscent to the corresponding DSC curve with a deviation from baseline between 225 and 300 K. In addition, the dielectric loss curve of HB1 and LB1 show a pronounced maximum at temperatures 290 and ~260 K, respectively, and are associated with the segmental dynamics at the chosen frequency (1.33 × 10^5^ Hz). The lower temperature for the LB1 is consistent with a lower glass temperature. This further shows that the initial step in the broad thermal transition seen in the DSC curve of LB1 (Figure 1) associates with the freezing of the segmental dynamics of the EB mid-block (*T*_g_). In addition, the peak height in |d*ε*′/d*T*| of LB1 is reduced with respect to HB1, revealing that only a fraction of the EB block is amorphous and is in agreement with the WAXD study.

### 3.6. Time Domain NMR

**Figure 8:**
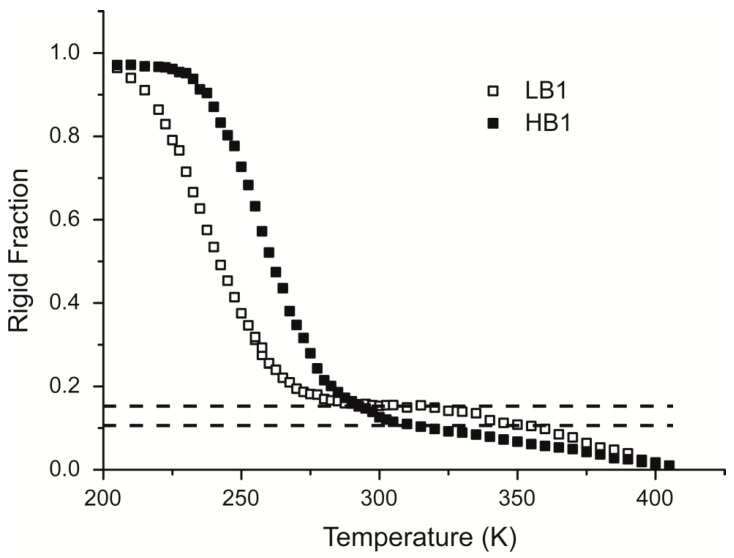
Temperature dependent measurement of the rigid fraction of LB1 and HB1 SEBS. Dashed lines represent the amount of rigid PS in the plateau region.

Measurement of rigid fraction *f_r_* was performed on FIDs acquired after the MSE refocusing block in all samples from 200 to 420 K at 2.5 K steps. The data were fitted with Equation (1), without constraining any of the parameters. As reported in the supporting information, the fitting was very good, and we did not observe the Abragamian function shape typical of ordered crystalline systems even at low *T*. A small correction (5–8%) was then added to the rigid fraction to offset the selective decay in the dead time with the given τ [32]. The results for samples HB1 and LB1 are represented on Figure 8. Data for other LB and HB samples are similar to the ones shown.

Unlike the DSC shown in Figure 2, the TD-NMR data show no difference between heating and cooling, since each point requires about 20 min, allowing for the system to equilibrate. At 200 K, in both HB1 and LB1 the rigid fraction is equal to unity, since below *T*_g_ of the EB block the entire sample is glassy. As the temperature reaches the onset of the DSC thermal events (213 K for LB1 and 221 K for HB1) the rigid fraction starts to decrease smoothly, until above 280 K where the EB block is mobilized. The remaining rigid fraction is due to the styrene block. From 280 K, the rigid fraction of LB1 remains constant for about 40 K, before declining again above 320 K. The *f_r_* measured in this plateau is attributed to PS blocks alone, since it is evaluated at 16%, very close to the predicted value of 18.6%. Further decline of the rigid fraction indicates a softening of the PS blocks at temperatures lower than the *T*_g_ of bulk PS, possibly due to the presence of interfaces [43]. This is in agreement with the absence of a detectable *T*_g_ in DSC for the rigid block. In the case of HB1, the glass temperatures of the EB block are higher, making a plateau harder to define. Still, in the range between 310 and 320 K, that is just before the onset of the PS glass transition, the rigid protons of HB1 are evaluated at 10%, very close to the predicted value of 11.2%.

For establishing the nature of the detected transition, the rigid fraction data obtained by TD-NMR were further analyzed using a model based on traditional free volume theory. As proposed by English and Inglefield [44], the Vogel–Tammann–Fulcher (VTF) equation was modified by introducing the excess free volume *h* = (1 − *y*) derived from the Simha and Somcynsky theory [45,46]: the hypothesis is that there exists some value of *h* below which the mobile population *f_m_* is zero and above which *f_m_* increases to a value of *f_m_*_0_ which would likely be one in a completely amorphous system. The equation used is the following:

$$f_{m}\left(T\right)=f_{m0}\left(1-\exp\left[-\frac{h\left(T\right)-h\left(T_{0}\right)}{h\left(T_{0}\right)}\right]\right)$$

where *h*(*T*) can be calculated from the Simha and Somcynsky equation-of-state and *f_m_*_0_ and *T_0_* are obtained by fitting the experimental values. Since the TD-NMR measurements are performed at ambient pressure, *h*(*T*) can be calculated only as a function of the reduced parameter T*. Using the modified VTF equation fittings of the temperature dependence of mobile fraction for LB1 and HB1 where obtained (supporting information). Interestingly, for LB1 *T*_0_ = 216 K and for HB1 *T*_0_ = 229 K were estimated. These values (“TD-NMR *T*_g_”) are in the vicinity of the *T*_g_ obtained by DSC. Therefore MSE TD-NMR measurements do not provide any hint of crystallization in the case of LB1. From this point of view, all samples behave as amorphous or weakly ordered polymers.

**Figure 9:**
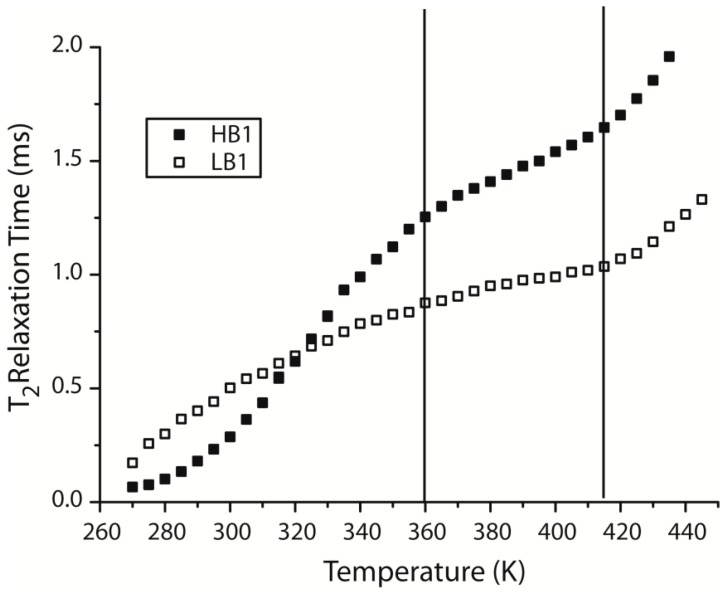
*T*_2_ as a function of temperature for reported SEBS samples, measured by the Hahn-Echo.

To further characterize the rubber chain mobility the Hahn-Echo pulse sequence with echo time τ > 50 µs was used to filter out the signal from the rigid phase [20]. Measured *T*_2_ values for LB and HB1 are shown in Figure 9.

Three different regions for the temperature dependence of *T*_2_ can be recognized: region I, from low temperatures to the *T*_g_ of S block (around 360 K). Region II, from the *T*_g_ of S block to 420 K where the rigid fraction reaches zero indicating full mobility also of the PS segments. Lastly, region III, is above 420 K.

The transverse relaxation behavior of rubbers is usually very complex, but it was proposed that even a single exponential fitting can provide an average assessment of polymer mobility that also relates well with mechanical properties. [47]. In ideal cases, considering a single phase amorphous polymer and following the Williams–Landel–Ferry (WLF) theory, an exponential equation was proposed for fitting *T*_2_ data as a function of the difference between glass temperature and observation temperature. SEBS samples show this trend in region III, where both blocks are well-above *T*_g_ and there are no rigid areas that can act as constrains for the free motion of the polymer chains. Region II cannot be interpreted in terms of WLF, since in this range both phases with their different dynamics contribute to the measured average *T*_2_ value. In region I, since the entire PS block is rigid and does not contribute to the T_2_, the WLF theory should be valid, but only HB SEBS show an exponential increase of about one decade between 280 and 340 K. LB systems show a dramatically different trend, which cannot be assimilated to an exponential growth. While a single exponential was usually an acceptable fitting for the Hahn-Echo data, we also applied more recent techniques specifically suited for characterizing network dynamics, namely the Multiple Quantum (MQ) NMR.

**Figure 10:**
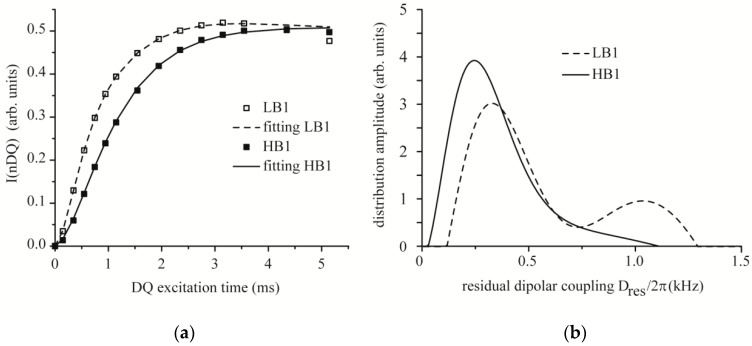
(**a**) nDQ data for sample LB1 and HB1 acquired at 353 K, with data fitting represented by dashed and solid lines, respectively. (**b**) Corresponding distributions of residual dipolar couplings.

The output of MQ measurements is shown in Figure 10a and is expressed in terms of normalized DQ (nDQ) intensity, that is the intensity of the MQ signal normalized against a reference curve and corrected for the presence of slow relaxing components such as sol fractions. It can be seen that the nDQ curve for both materials tends to a value of 0.5 at high evolution times, as predicted for crosslinked systems. The steeper slope of the initial part of the buildup indicates, intuitively, that motions in LB1 display a higher order parameter, and a calculation of the actual distribution was performed as in ref [34] and shown in Figure 10b for LB1 and HB1. Results in Figure 10 are representative for the rest of HB and LB samples.

The distribution of dipolar couplings of LB samples are shifted towards higher values, indicating that these materials have the highest average order parameter at 353 K despite being further away from *T*_g_. The bimodal distribution in LB1 is usually associated to heterogeneities, such as the presence of clusters due to micro-separation in regions of different polarity as in the case of substituted EPDM rubbers [48]. Here, it indicates a separation of the mobile phase in zones of distinct dynamic order. HB1 samples instead present the monomodal distribution usually found in rubbers [27], with only a tail extended towards higher values that can be associated to a mobility gradient in the immediate vicinity of the PS block.

## 4. Discussion

SEBS samples with low butylene content show the broad endothermic feature described in literature (Figure 1), whereas the ones with high butylene display a classical calorimetric glass temperature. Other features, such as polymer molecular weight or the nanodomain morphology seem to play little or no role. Different experimental approaches produce a conflicting view on whether this event is due to crystallization of ethylene segments in the EB phase. For comparison, we note that it was demonstrated using precisely branched PEs that as low as six consecutive ethyl units are sufficient for crystallization [49]. Furthermore, crystallization under strain has been directly demonstrated in SEBS with less than 50% butylene by Lesser et al. [11]. However, it should be noticed that the DSC endotherm of LB SEBS is similar to the behavior reported by Crist and coworkers [50] for hydrogenated polybutadiene (HPB), equivalent to the central block of SEBS. Crist, on the basis of the theory of Flory for crystallization of random copolymers, proposed an analytical equation for calculating the degree of crystallinity as a function of the comonomer content and of thermodynamic and geometric properties of the polyethylene crystals. The temperature derivative of the crystallinity fraction is closely related to the DSC trace. As the mole fraction of the non-crystallizable monomer increases, the melting temperature of PE crystals decreases and the melting peaks become broader. For example, while a HPB with 2.6 mol % of butylene melts quite sharply at 394 K, a HPB with 21.2 mol % butylene starts to melt below room temperature and is fully molten at 334 K [51]. Analogous results can be found in Carella et al. [52], where a melting temperature of 301 K is reported for a HPB containing 27.0 mol % of butylene.

**Figure 11:**
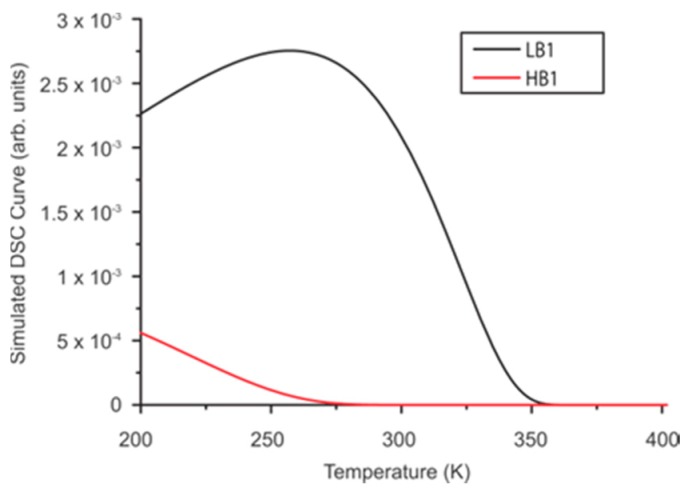
Simulated DSC curves for LB1 and HB1 central blocks. The *y* axis represents the incremental fraction of melting PE.

On the basis of the equations proposed by Crist and coworkers, we calculated the predicted DSC trace corresponding to the EB block, Figure 11. In the limit of low temperature, the calculated DSC becomes unphysical because it neglects real polymer kinetics like the glass temperature itself, but the breadth of the transition and the melting temperature are confirmed by the literature as discussed above. The prediction is that HPB copolymers with the same composition of the poly(ethylene-co-1-butylene) block of SEBS LB1 and LB2 would present “final” melting temperatures not less than 325 K. In SEBS, the endotherm extends only to 300 K, with a difference of more than 20 K.

Moreover, the temperature evolution of rigid phase detected by TD-NMR in LB SEBS provided no direct evidence of crystallization, being compatible with the behavior of an amorphous phase undergoing liquid-to-glass transformation, as in HB SEBS. Instead, the *T*_2_ relaxation time of the mobile phase detected through the Hahn-Echo sequence and plotted in Figure 9 indicates a qualitative difference in the range below 360 K between LB and HB SEBS. The mobile phase of the HB sample has an exponential increase of the *T*_2_ with temperature that is typical of rubber materials, while the LB sample tends to an asymptotic value. On the other side, WAXS provided direct proof of crystallization in the LB samples below the DSC detected transition, even if that crystallization is highly defective.

A model that can explain, at the same time, DSC, X-ray, DS and NMR results invokes the existence of a rotator phase in the LB samples between 300 and 360 K, which evolves in a weakly crystalline system at lower temperatures. A rotator phase has been demonstrated [15] in random EP copolymers, but in that case it was clear that the methyl side group could also be included in the pseudocrystalline phase. This is not usual for longer side groups such as the butyl side chains of SEBS: it was observed directly that side chains in poly (ethylene-*co*-octene) systems are selected out of the crystal phases [53]. One must consider the effect of the rigid constraints provided by the nanophase separated PS blocks, as demonstrated by ^13^C MAS NMR spectra reported in Figure 5 and clearly shown by SAXS. Such an intimate blending influences the stability of the phases formed in the polymer. In fact, systems where PS lamellae are separated by less than 20 nm can host polymer structures that are otherwise metastable, transient or inaccessible [14,54].

However, SEBS is not strictly a constrained system, since in all cases the mobile phase is continuous (SAXS). Moreover, the length of chains between the blocks seen in Table 1 is several times larger than the typical *M*_e_ (molecular weight between entanglements) value for EB [55]. Thus, several entanglements are predicted between the hard constraints created by the polystyrene part, and the system is locally similar to an entangled melt or non-vulcanized rubber. Furthermore, the X-ray analyses have shown non-equilibrium structures in the as-cast films that reach the equilibrium nano-domain morphologies only after heating to 200 °C and annealing for long times. On the other side, tethering on both ends of the EB chains prevents reptation, and the modification of topology of the EB phase takes place mostly by complex cooperative motions that end up in changing the position of the entanglements. This process is slow enough to be on a different time scale than the motions probed by MQ NMR, at least to the extent that the nDQ signal achieves the 0.5 value typical of plateau values in the polymer self correlation function.

We propose that, upon cooling from melt state, the SEBS with the lower butylene content (i.e., LB) forms a rotator phase that produces the higher slope in the nDQ curve and the corresponding peak at high *D*_res_ values. The glass temperature of the EB part is then shifted to slightly lower temperatures (10 K) as compared to the HB family. On the other side, the high butylene content samples cannot present this phase because the steric hindrance is too high.

## 5. Conclusions

In this work we characterized the EB phase of several nanophase separated copolymers of SEBS, a relevant family of industrial thermoplastic elastomers that display calorimetric phase transitions that are not fully understood. The most impacting features on the organization of this phase are the presence of a certain amount of side chains that cannot be accommodated in a PE crystal lattice, but also the presence of tethering to the PS blocks formed by nanophase separation. While the copolymers rich in butylene (~50%) behave like locally amorphous systems in terms of all applied techniques (DSC, DS, WAXS), the samples with low (~20%) butylene content display more complex thermodynamic and structural properties, including a lower liquid-to-glass temperature and weak crystallization up to 300 K. This is different not only from the bulk EB, where samples with 16% butylene content have less than 2% crystallinity [40] but also from the much more similar HPB [51]. The latter exhibit a “low” glass temperature some 10 K above the LB family. Using Time Domain NMR, we could explain the observed phenomena with the formation of rotator PE phase between 300 and 350 K. The formation of such phases in ethylene-propylene copolymers is well-known and reflects the possibility to accommodate the short methyl side groups in the loosely organized pseudo crystalline lattice, which is normally not possible in the presence of bulkier ethyl groups. The origin of the rotator phase—that exists only in the LB family—can be associated with the longer characteristic spacing of the nanodomains—as demonstrated by SAXS—despite the lower molecular weights of the copolymers. Locally extended polymer segments in LB can promote the nucleation and subsequent growth of the rotator phase. Such a phase will be prone to annealing/thermal history as experimentally observed (SAXS).

## Figures and Tables

**Table 1:** *M*_n_ and polydispersity index (PDI) are obtained from size exclusion chromatography SEC. The number of units is estimated from the total *M*_n_ and the ratio between the integrals of the different repeating units measured by NMR.

	Styrene mol %	Butylene mol %	Ethylene mol *	*M*_n_ × 10^4^	PDI	PS units (per block)	EB units
LB1	18.6	23.8	76.2	6.0	1.07	86	752
LB2	18.6	23.0	77	7.9	1.08	113	990
LBS	27.7 ^§^	21.8	78.2	10.6	1.13	212	1105
HB3	11.5	57.8	42.2	10.0	1.06	93	1439
HB1	11.2	67.4	32.6	10.2	1.09	93	1476
HB2	7.1	64.3	35.7	15.9	1.08	95	2486

* % relative only to the mid block; ^§^ 37% of total styrene is located in the mid block.

**Table 2:** Thermodynamic and structural data of the copolymers based on DSC and X-ray diffraction studies. *T*_g_ and end of transition are relative to DSC heating run. The beginning of the transition is considered as *T*_g_ for the samples who display it. The upper limit of the transition is given when present.

Sample	Lower *T_g_* (K)	Upper bound of transition (K)	Nanodomain morphology *^a^*	Characteristic spacing, *d* (nm) *^b^*
LB1	213	301	lam/hcpc	35.7 (lam) 25.2 (hcpc)
LB2	211	300	hcpc	34.5
LBS	226	298		
HB3	218	-		
HB1	221	-	hcpc	24.3
HB2	225	-	bcc	23.9

*^a^* Obtained on slow cooling from 473 K. *^b^* Lamellar: *d* = 2π/q*, hcpc: *d* = (2π/q*)(4/3)^1/2^, bcc: *d*_0_ = *d*(3/2)^1/2^ and R_PS_ = (*f*_PS_*d*_0_^3^/π3^1/2^)^1/3^.

## Supplementary Materials

The supplementary materials are available online at http://www.mdpi.com/2073-4360/10/6/655/s1.
