# Supplementary material for: Local Order and Dynamics of Nanoconstrained Ethylene-Butylene Chain Segments in SEBS
Source: Polymers (Basel). 2018 Jun 11;10(6):655. doi: 10.3390/polym10060655 (PMC6404420; doi:10.3390/polym10060655)
Supplement: Supplementary file 1 [file polymers-10-00655-s001.pdf]

Local Order and Dynamics of Nanoconstrained Ethylene-Butylene Chain Segments in SEBS – Supporting Information

Michele Mauri,† George Floudas,‡,┼ and Roberto Simonutti*†

† Department of Materials Science, University of Milano Bicocca, via R. Cozzi 55, 20125 Milan, Italy; Tel.: +39 0264485132; Fax.: +39 0264485403.

‡ Department of Physics, University of Ioannina, 45110 Ioannina, Greece

┼ Max Planck Institute for Polymer Research, Ackermannweg 10, D-55128, Mainz, Germany

The following supporting information is provided:

1) Example of Size Exclusion Chromatography
2) Example of High Resolution ^{13}C NMR
3) DSC of high butene content sample
4) Additional details on SSNMR
5) Additional details on TD-NMR
6) Example of TEM
7) SAXS

1. Size Exclusion Chromatography (SEC)

Gel Permeation Chromatography was performed with a Waters 1515 Isocratic HPLC pump using solutions of polymer in THF with a concentration of 4 mg/2mL (0.2% wt). Solubility at this concentration was very good. THF flow was 1mL/min and temperature was maintained at 298 K. Detection was provided by using a refractive index detector Waters 2414. Figure S1 depicts the result for sample LB1, whence the molecular weight can be obtained as well as the polydispersity index.

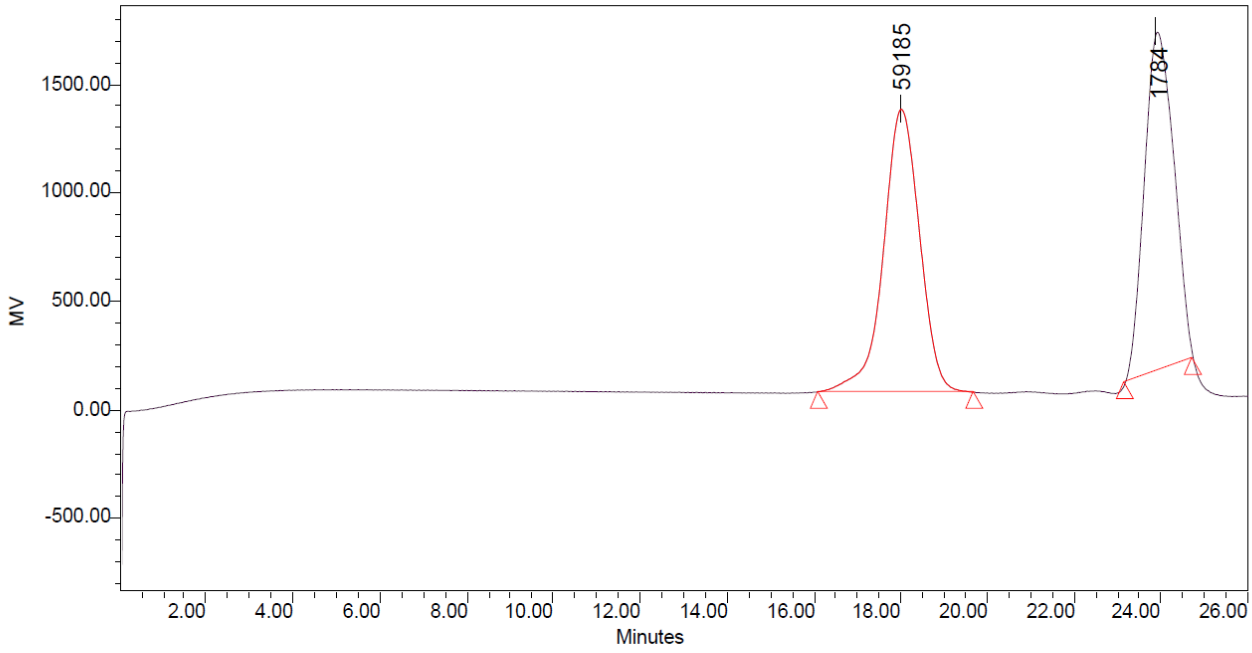

Figure S1: SEC of sample LB1, with peaks corresponding to polymer LB1 and THF (left to right).

Samples HB1 and HB3 also present a secondary (less than 10% integral) peak associated to a smaller weight component, possibly due to incompletely formed SEBS chains.

2. High resolution NMR

As exemplified by Figure S2, the spectrum of such complex terpolymer has important splitting phenomena due to different microstructural environments.

Region (ppm)	Assignment	Integral
145	Quaternary C (PS)	4.47
125-128	Phenyl C (PS)	
76-77	Solvent Residual	NA
25-40	Aliphatic carbons (PS and EB)	10.6
10-11	Methyl C (B unit within EB)	1

Methyl signals are univocally associated to carbon B_2 of side chains associated to butene monomer, and resonate around 11 ppm. The splitting of that signal is due to differences between isolated side chains, and side chains that are located besides others.

While the methyl signals are associated to the But comonomers, as indicated in table ST1, resonances at 145 and around 126 ppm are unambiguously associated to the PS block. On the other side, the region between 20 and 40 ppm contains aliphatic signals that could originate from the main chain carbons of each of the blocks. Simple mathematical considerations allow to use the integrals to obtain both the relative amount of PS and EB, and the amount of EB within the mid block, reported in TABLE 1 of the main paper.

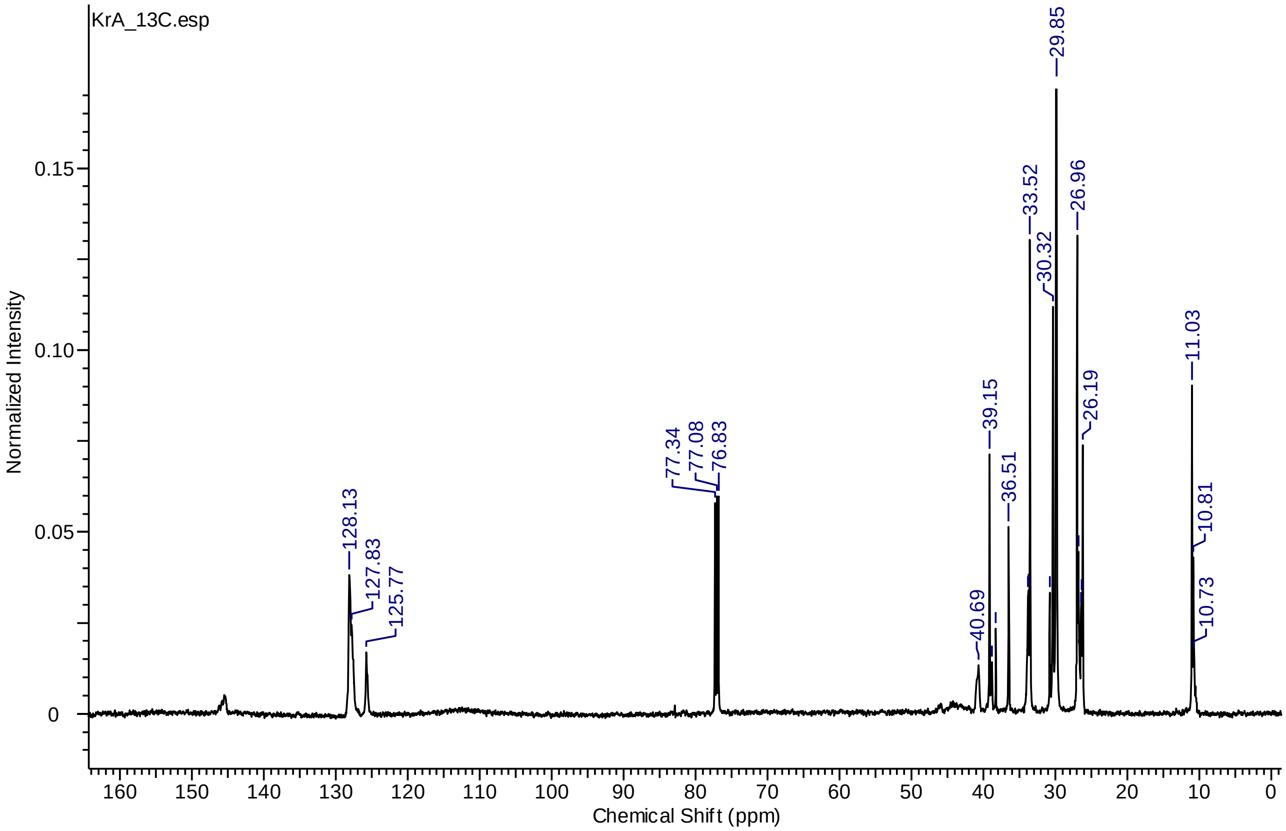

Figure S2: ^{13}C NMR spectrum of sample LB1, dissolved in $CDCl_3$.

3. Differential Scanning Calorimetry (DSC)

The DSC was calibrated for temperature and heat flow using indium, tin and nonane. Experiments were always performed at 10 K/min. The oven was purged with a dry N_2 flow of 80 mL/min. Experiments were performed on aliquots of around 5 mg in 40 µL pans made of aluminum. The temperature cycle started from 173 K and samples were heated to 403 K at a rate of 10 K/min. Then all samples were cooled back to 173 K and heated again to 403 K. This cycle is performed to equalize the thermal history of the samples, since the temperature is well over the Tg of the PS blocks.

Figure S3 shows the DSC analysis of a sample with high butene content (HB1). The glass transition temperature is very similar between the first and second heating cycle. Comparison of heating and cooling curves does not show the peak associated to crystallinity that is instead evident in the DSC of sample LB1 presented and discussed extensively in the main text (Fig. 2).

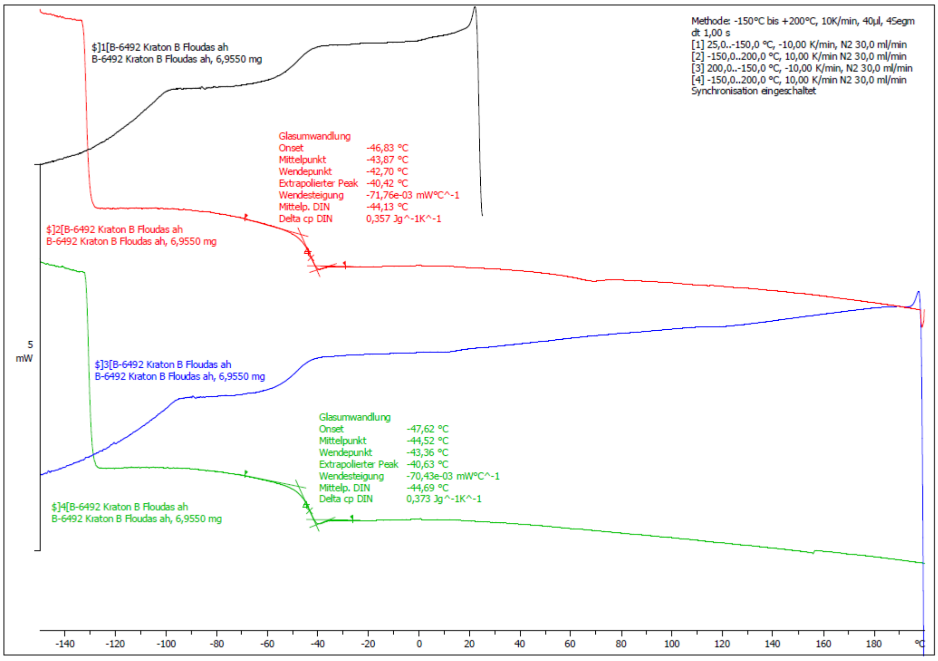

Figure S3: ^{13}C NMR spectrum of sample LB1, dissolved in $CDCl_3$.

4. Additional Details on SSNMR

The ramp is obtained changing the strength of the proton RF from 80 kHz at the beginning of the contact time to a value of 60 kHz at the end of the cross-polarization period in 100 steps. The 90° pulse for proton was 2.9 µs. Cross-polarization (CP) MAS experiments were performed using a recycle delay of 10 s and a contact time of 2.5 ms. Single pulse excitation (SPE) experiments were run using a recycle delay of 20 s.

5. TD-NMR details

The MSE sequence used for the measurement of rigid fraction is depicted in Figure S4 and is able to refocus the ^{1}H multinuclear dipolar interaction of the rigid solid state[1] with only minimal loss due to actual relaxation during the echo time τ. This loss was evaluated by performing several experiments with increasing τ and then back extrapolating to τ = 0, as in literature[2]. The MSE refocusing scheme is efficient either in the slow- or the fast motion limit, but is impeded when molecular motion takes place on the time scale of the refocused interaction (approximately the reciprocal of the dipolar coupling) and of the echo (both ~50 µs). The efficiency loss of MSE can be relevant 30-40 K above T_g, for this reason at critical temperatures MSE data were complemented with the analysis of unrefocused FIDs recorded at the same temperature[3].

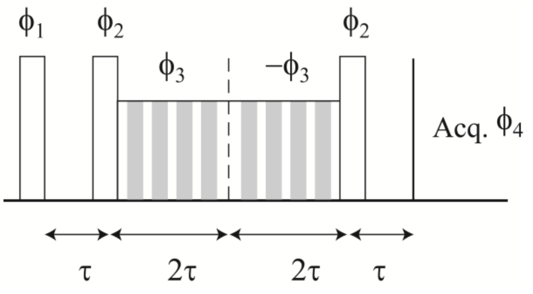

Figure S4: π/2 pulse on the left, followed by a Magic Sandwich Echo (MSE) refocusing block. All pulses are π /2, refer to Ref. Error! Bookmark not defined. **for details on pulse phase and interpulse spacing. The 8 pulses in grey can be iterated n times to increase sequence duration. Time span τ must be greater than the receiver dead time.**

The measurement of rigid fraction is performed at stepwise increasing temperature. A typical example of the resulting FIDS is reported in the left side of Figure S5. Each acquired FID be separated by decomposition of the initial 200 µs of the FID in rigid (f_r) and mobile (f_m) fractions as described in the main text (Eq. 1). In this model, the rigid fraction is represented by a Gaussian function, and the mobile fraction by a stretched exponential (Weibullian) function, resulting in very good fits such as the one reported in the right side of the same Figure S5.

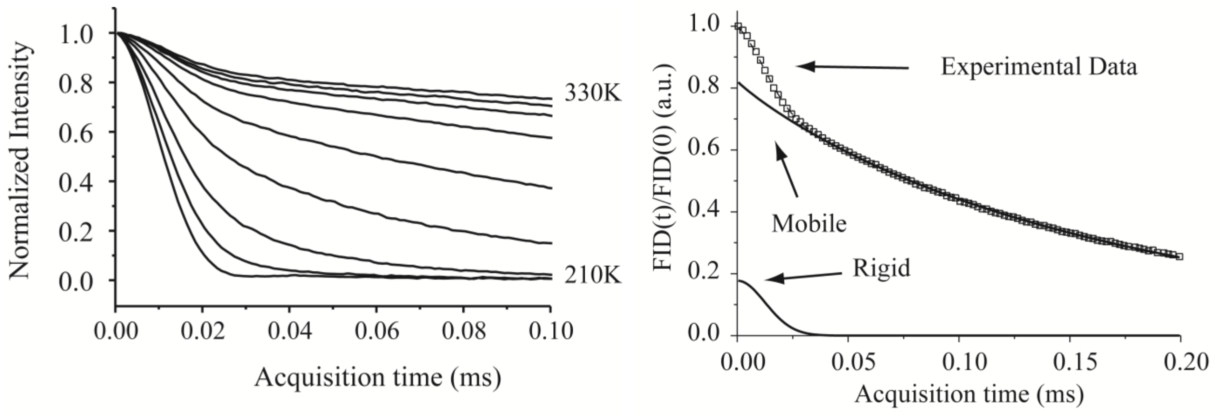

Figure S5: on the left, FIDs of sample HB1 acquired at 15K increments after application of the MSE block. Initial intensities were normalized to 1. Experiments were performed at steps of 2.5 K, but only some are plotted for the sake of clarity. On the right, fitting of HB2 at 273. Solid lines indicate the Gaussian and Weibullian functions. The fitting sum function is practically coincident with the experimental data.

As depicted in Figure S6, and discussed in the main text, the rigid fraction extracted from each FID is plotted against temperature and fitted with eq. (2) in order to obtain a time domain NMR glass transition temperature (TD-NMR T_g). In the case of sample LB1 it is equal to 221K. Notice that the function does not tend to unity due to the presence of the second (PS) block. Moreover, the fitting is not perfect, another indication that the phenomenon is not completely represented by a pure glass transition but contains more complex rearrangement of the polymer chains.

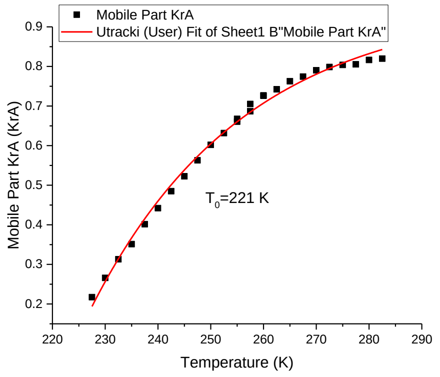

Figure S6: rigid fraction of sample LB1 measured at 2.5 K intervals and evaluated by FID decomposition after application of MSE block. The data are fitted with eq (2).

Details of MQ (Baum Pines) sequence

The Baum Pines sequence used for determining the residual dipolar coupling and its distribution is represented in figure S7, with an example of the data in the following Figure S8.

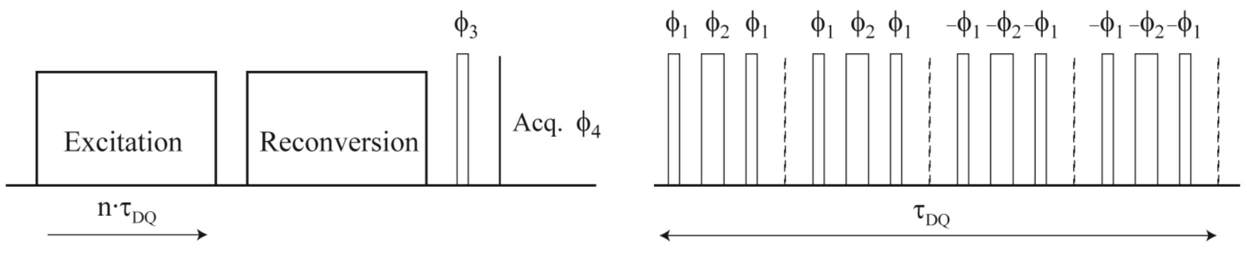

Figure S7: (left) conceptualization of the Baum Pines sequence, emphasizing the excitation time. Excitation is composed of an even number n of cycles with duration τ_{DQ}, described in detail in the right part of the figure. Depending on receiver phase, DQ or Ref signal is acquired.

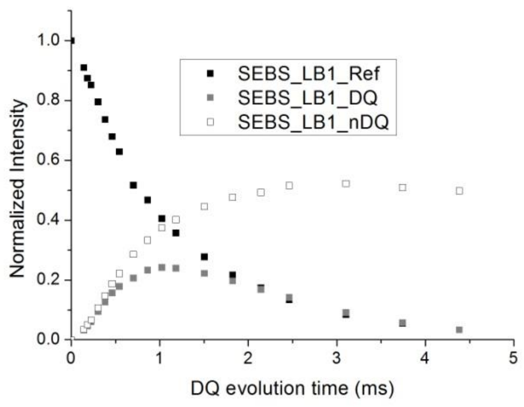

Figure S8: DQ signal starts from zero and builds up with DQ excitation time. The apparent decay is due to relaxation as seen in the decay of the Ref signal. Normalizing the DQ signal against the total (DQ+Ref) intensity produces the nDQ curve that contains the relevant information. This example is relative to LB1, measured at 353K. The fitting of the nDQ curve and the resulting D_{res} distribution are the ones depicted in the main text.

Details of Hahn Echo fitting

The Hahn Echo sequence is a mainstay of rubber investigation. Figure S9 depicts a typical experiment, and the effectiveness of the monomodal fitting.

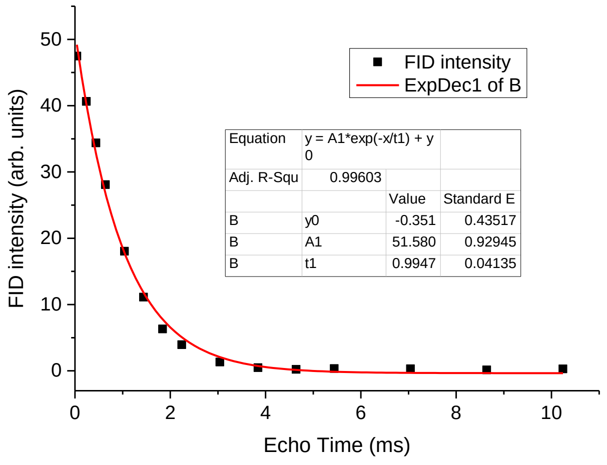

Figure S9: Hahn Echo experiment performed on sample HB1 at 343K.

6. TEM

An example of nanostructure was imaged using a Tecnai G2F20 microscope (FEI Company, the Netherlands) equipped with a Schottky Field Emission electron source, a US1000 2kx2k Gatan CCD camera and operating at an acceleration voltage of 200 kV. The sample was cast from toluene and stained with uranyl acetate. The lamellar structure, corresponding to nanophase separation, is apparent from Figure S10.

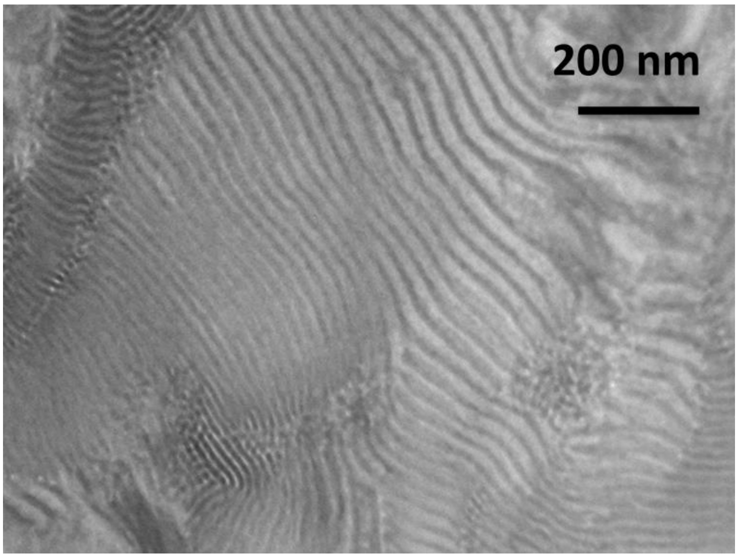

Figure S10: TEM of sample LB1 cast from toluene, with evidence of phase separation (lamellar structure). Due to the staining procedure the black one is PS.

7. SAXS

Figure S11 depicts the variable temperature SAXS measurements of the low butene sample LB1, for comparison with the HB discussed in the main text. Annealing causes reversible and irreversible changes on the nano-domain morphology. The dependence of the first and higher order reflections following annealing at 473 K indicates shrinkage upon heating. The final morphology is a mix between lamellar (lam) and hexagonally closed packed cylinders (hcpc)

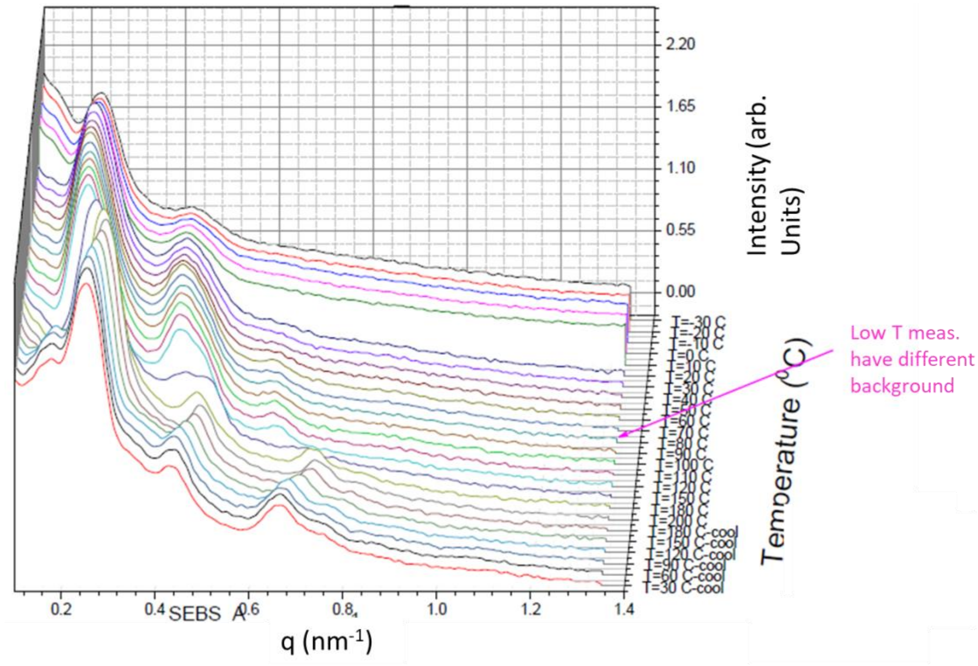

Figure S11: SAXS intensity profiles for sample LB1, with low butene content in the mid block, shown for different temperatures on heating and subsequent cooling.

References

1. Maus, A.; Hertlein, C.; Saalwächter, K. A robust proton nmr method to investigate hard/soft ratios, crystallinity, and component mobility in polymers. *Macromolecular Chemistry and Physics* **2006**, *207*, 1150-1158.
2. Mauri, M.; Thomann, Y.; Schneider, H.; Saalwächter, K. Spin-diffusion nmr at low field for the study of multiphase solids. *Solid State Nuclear Magnetic Resonance* **2008**, *34*, 125-141.
3. Papon, A.; Saalwachter, K.; Schaler, K.; Guy, L.; Lequeux, F.; Montes, H. Low-field nmr investigations of nanocomposites: Polymer dynamics and network effects. *Macromolecules* **2011**, *44*, 913-922.
